# Hybrid immunity following SARS-CoV-2 infection and vaccination is associated with more sustained antibody levels, and lower reinfection risk

**DOI:** 10.1038/s41598-026-55396-x

**Published:** 2026-05-29

**Authors:** Naim Che-Kamaruddin, Jefree Johari, Hasmawati Yahaya, Nurhafiza Zainal, Huy Nguyen, Robert D. Hontz, Andrew G. Letizia, Sazaly AbuBakar

**Affiliations:** 1 Tropical Infectious Diseases Research and Education Centre (TIDREC), Higher Institution Centre of Excellence (HICoE), Universiti Malaya, Kuala Lumpur, 50603 Malaysia; 2 Department of Medical Microbiology, Faculty of Medicine, Universiti Malaya, Kuala Lumpur, 50603 Malaysia; 3 U.S. Naval Medical Research Unit INDO PACIFIC, Singapore, Singapore

**Keywords:** Immunology, Microbiology

## Abstract

A decline in antibody levels against SARS-CoV-2 over time is expected and may lead to breakthrough infections (BTI; infection occurring after vaccination) and reinfections (infection occurring after recovery from a prior SARS-CoV-2 infection). This 12-month longitudinal study investigated the effects of antibody kinetics following SARS-CoV-2 vaccination and infection on the timing and likelihood of BTI or reinfection. Serum IgM, IgA, IgG, and neutralizing antibodies (NAb) against the ancestral SARS-CoV-2 variant were measured in a prospective cohort of 267 participants in Malaysia from March 2021 to May 2023, stratified by the order and timing of SARS-CoV-2 infection and vaccination relative to study enrollment. IgG levels following SARS-CoV-2 infection in individuals previously vaccinated with CoronaVac were higher up to Day 60 compared with those who received BNT162b2 or ChAdOX1 as their primary vaccination; however, antibody levels across vaccine platforms were not statistically different at six months and beyond post-vaccination. The risk of BTI was similar across vaccine platforms, whereas individuals with a history of SARS-CoV-2 infection prior to vaccination showed the lowest subsequent reinfection risk. Breakthrough infections in vaccinated individuals were associated with more sustained IgA, IgG, and NAb levels compared with vaccinated individuals without infection. Our results demonstrate that exposure sequence, rather than vaccine platform alone, is a key determinant of long-term antibody durability and reinfection risk, providing clinically interpretable evidence to inform vaccination strategies in the endemic phase of SARS-CoV-2.

**Supplementary Information:**

The online version contains supplementary material available at 10.1038/s41598-026-55396-x.

## Introduction

The nationwide Coronavirus Disease 2019 (COVID-19) vaccination campaign in Malaysia in 2021 resulted in a large portion of the population developing vaccine-induced immunity against the Severe Acute Syndrome Coronavirus 2 (SARS-CoV-2), contributing to population-level protection^1^. Despite the high immunization coverage, a significant number of individuals were subsequently infected with SARS-CoV-2^2^. The increasing incidence of breakthrough infections (BTI) or reinfections presents challenges in accurately interpreting seroprevalence to determine correlates of protection against SARS-CoV-2^3^. The occurrence of BTI cases has also impacted the dynamics of person-to-person transmission^4^, raising concerns about the long-term effectiveness of immunity following booster vaccination doses.

The long-term durability of antibodies is influenced by various exposures to SARS-CoV-2 through vaccination, natural infection, or hybrid immunity^4–6^. These exposure histories contribute to variability in the magnitude and persistence of humoral immune responses^7^, particularly as SARS-CoV-2 becomes endemic and new variants continue to emerge^2,4,8^. Few longitudinal studies have simultaneously evaluated antibody kinetics, neutralization capacity, and infection risk across multiple real-world exposure sequences within a single cohort, limiting the interpretability of immune durability in the post-vaccination era. Therefore, our study aims to explore the differences in antibody levels and durability influenced by SARS-CoV-2 exposures, with a focus on evaluating risk and duration using BTI or reinfection as end points. Understanding the protection offered by various exposures is crucial for understanding the dynamics of humoral responses with diverse immunity against SARS-CoV-2^9^. The findings of this study may serve as a guide for developing future countermeasures aimed at mitigating the threats of new variants that may pose an increased risk to global public health^10–12^.

## Methods

### Ethical clearance

This study was conducted in accordance with the Declaration of Helsinki, and the protocol was approved by the Universiti Malaya Medical Centre-Medical Research Ethics Committee (MREC-UMMC SID: 2021226-9886). All participants provided informed consent for inclusion before they participated in the study.

### Study design and participant recruitment

Our longitudinal, prospective observational study collected serum samples from 400 participants over a period of 12-month in Kuala Lumpur, Malaysia. The participants were categorized into five groups according to their vaccination status and history of COVID-19 (Table 1). The history of SARS-CoV-2 exposure among the participants was determined by (1) certificates of COVID-19 vaccination; (2) confirmation of SARS-CoV-2 infections via Polymerase Chain Reaction (PCR) and/or Rapid Test Kit (RTK); or (3) a significant four-fold rise in IgM and/or IgG levels relative to previous sampling timepoints, as elaborated in our study protocol article^13^.

**Table 1:** Study groups.

Study groups	Definition
1-E/VAC(BNT)/COVID-ve (*n* = 52)	SARS-CoV-2-naïve participants enrolled ***(E)***, received the primary series of BNT162b2 vaccination ***(VAC(BNT))***, and did not experience a breakthrough COVID-19 during the 12-month follow-up period ***(COVID-ve)***.
2-E/VAC(BNT)/COVID+ve (*n* = 85)	SARS-CoV-2-naïve participants enrolled ***(E)***, received the primary series of BNT162b2 vaccination ***(VAC(BNT))***, and had a breakthrough COVID-19 during the 12-month follow-up period ***(COVID+ve)***.
3-VAC(BNT)/COVID+ve/E (*n* = 62)	Participants received the primary series of BNT162b2 vaccination ***(VAC(BNT))***, subsequently developed a breakthrough COVID-19 ***(COVID-19 + ve)***, and enrolled in the study within 30 days of infection ***(E)***.
4-VAC(non-BNT)/COVID+ve/E (*n* = 39)	Participants were vaccinated with a primary series other than BNT162b2 ***(VAC(non-BNT))***, subsequently developed a breakthrough COVID-19 ***(COVID+ve)***, and enrolled in the study within 30 days of infection ***(E)***.
5-COVID+ve/E/VAC(any) (*n* = 29)	Unvaccinated participants who had a COVID-19 ***(COVID+ve)***, subsequently enrolled in the study within 30 days of infection ***(E)***, and received any COVID-19 vaccination during the 12-month follow-up period **(VAC(any))**.

Abbreviations: SARS-CoV-2, Severe Acute Respiratory Syndrome Coronavirus 2; E, study enrollment; VAC(BNT), vaccinated with BNT162b2; VAC(non-BNT), vaccinated with a COVID-19 vaccine other than BNT162b2; VAC(any), vaccinated with any type of COVID-19 vaccine; COVID-ve, negative for COVID-19; COVID+ve, positive for COVID-19; “/“ delineates sequential events in chronological order.

The recruitment process utilized a non-probabilistic method, combining both convenience and volunteer sampling methods. Participants from groups 1-E/VAC(BNT)/COVID-ve and 2-E/VAC(BNT)/COVID+ve provided their baseline serology to assess pre-vaccination antibody levels (Cohort-1). Participants received the first and second doses of the primary BNT162b2 vaccination 21 days apart. Participants from groups 3-VAC(BNT)/COVID+ve/E, 4-VAC(non-BNT)/COVID+ve/E, and 5-COVID+ve/E/VAC(any) provided their first serum sampling within 30 days of post-symptom onset of COVID-19 (Cohort-2). The participants were followed up on a weekly, monthly, or tri-monthly basis for up to 360 days (Supplementary Fig. 1). Follow-up timepoints were calculated in individual days, with samples collected between March 2021 and May 2023. Each designated timepoint had a window period of ± 7 days.

For comparative analyses across study groups within each antibody class, overlapping timepoints were used (Supplementary Fig. 1). The timepoints referred to as Day-14, Day-30, Day-90, Day-180, Day-270, and Day-360 after the first BNT162b2 vaccine dose or after the onset of symptoms. These timepoints were contemporaneous in all study groups following SARS-CoV-2 exposures. Following recruitment, the participants were regularly monitored to document any SARS-CoV-2 test results (either from RTK or PCR confirmation, as required by the Ministry of Health Malaysia) and COVID-19 vaccination/boosters throughout the 12-month follow-up period. During the follow-up period, receipt of COVID-19 booster vaccinations was not uniform across participants and occurred in accordance with national vaccination policies in Malaysia. Booster vaccination status, including vaccine type and date of administration, was documented through vaccination certificates and participant self-report. Booster doses were treated as subsequent SARS-CoV-2 antigen exposures rather than as a primary stratification variable, as the timing and uptake varied across individuals and study groups.

### Enzyme-linked immunosorbent assays (ELISAs)

Four commercially available ELISA kits were utilized to measure SARS-CoV-2- specific antibodies, including immunoglobulin M (IgM) (WANTAI, Beijing Wantai Biological Pharmacy Ent.), immunoglobulin A (IgA), immunoglobulin G (IgG), and neutralizing antibodies (NAb) (EUROIMMUN, Lubeck, Germany). All ELISA assays employed antigens derived from the ancestral SARS-CoV-2 variant (Wuhan-Hu-1).

IgM antibodies targeted the receptor-binding-domain (RBD) of the spike (S) protein. IgA and IgG antibodies were measured using ELISAs targeting the S1 subunit. Neutralizing antibody activity against the ancestral SARS-CoV-2 variant was assessed using a surrogate Virus Neutralization Test (sVNT), a competitive ELISA that measures inhibition of the spike RBD and the human angiotensin-converting enzyme 2 (ACE2) receptor interaction without the need for live virus or BSL-3 facilities. Briefly, serum samples were incubated with HRP-conjugated RBD, followed by transfer to ACE2-coated microplates. Neutralizing capacity was expressed as percentage inhibition of RBD-ACE2 binding.

ELISA assays were performed on serum samples collected at each timepoint in strict accordance with the manufacturer’s protocols. Optical density (OD) values were measured at 450 nm with a reference wavelength of 650 nm. The reported sensitivities and specificities of the commercial ELISA kits ranged from 86.1% to 96.9%, and 98.3% to 99.8%, respectively.

The positivity threshold for RBD-specific IgM was defined as an OD value of 0.105. For S1-specific IgA, semiquantitative results were expressed as OD ratios calculated using the sample OD relative to the provided calibrator, with a positivity cut-off value of 1.1. Quantitative determination of S1-specific IgG was expressed in World Health Organization (WHO) international binding antibody units per mL (BAU/mL) using manufacturer-provided standards, with a positivity cut-off value of 35.2 BAU/mL. Serum NAb activity was expressed as the percentage of inhibition of RBD-ACE2 binding.

### Statistical analyses

For comparative analyses, Chi-square and Fisher’s exact test were employed for categorical variables, while the Mann-Whitney U test was performed for non-parametric comparisons of continuous variables between groups. Spearman correlation test was used to explore the correlation between variables and the non-parametric data. Linear regression lines were used to estimate the best fit datapoints in scatterplots. We employed a log-transformation to normalize IgG levels (BAU/mL) due to varying antibody ranges among participants, ensuring consistent distribution across study groups (when appropriate).

The Generalized Estimating Equation (GEE) was used to estimate the changes in the longitudinal distribution of antibodies across all participants. The 1-E/VAC(BNT)/COVID-ve group was used as the reference group in this study to compare the repeated antibody measurements in the GEE analysis. In the GEE model, a positive coefficient indicates that the antibody levels in a particular group are higher than in the reference group, while a negative coefficient indicates that the average antibody levels in that group are lower than those in the reference group.

The Kaplan-Meier estimator was used to calculate cumulative “time-to-infection” rates for different timepoints after vaccination (day of first dose, day of second dose, and Day-15 post-second dose), to provide information on the temporal dynamics of SARS-CoV-2 breakthrough infection risk post-vaccination. The incidence and latency to BTI were compared by categorizing the participants based on their IgG levels following the primary BNT162b2 vaccination series in the group that had both pre-vaccination and BTI data. The categorization was determined by employing the lower 25% and upper 75% quartiles as delineating points. Specifically, antibody levels that fell below the 25th percentile were classified as “Low”, while levels that surpassed the 75th percentile were classified as “High”. The intermediate range, which comprised the middle 50% of the distribution was categorized as “Moderate” IgG levels. The IgG levels were also compared between the groups on Day-14 (primary response post-SARS-CoV-2 antigen exposure), Day-37 (complete vaccination period), and Day-90 (early waning antibody period) following first BNT162b2 dose, and the differences were analysed using a Chi-square test.

The term “seroconversion” was used to describe the increase in antibody levels and “seroreversion” described the decrease in antibody levels.

Statistical analyses were performed using R software version 2023.12.0 + 369. Statistical significance was determined at 95% confidence interval, with *p*-values of 0.05 or less were considered as statistically significant.

## Results

### Study cohort description

Out of 400 participants enrolled in the study, 267 completed the 12-month longitudinal serum sampling and were included in the analysis. A total of 137 (51.3%) participants who tested serologically naïve to SARS-CoV-2 anti-S1 IgG were enrolled for baseline serum sample before receiving the primary series of BNT162b2 vaccination (i.e., groups 1-E/VAC(BNT)/COVID-ve and 2-E/VAC(BNT)/COVID+ve), while 130 (48.7%) participants were enrolled following documented SARS-CoV-2 infection within 30 days of infection, regardless of vaccination status (i.e., groups 3-VAC(BNT)/COVID+ve/E, 4-VAC(non-BNT)/COVID+ve/E, and 5-COVID+ve/E/VAC(any)). The median age of participants was 36 years old, ranging from 15 to 65 years old, with the majority being females (*n* = 167, 60.7%).

The primary series of COVID-19 vaccinations was predominantly composed of BNT162b2 vaccines (*n* = 220, 82.4%), with a smaller proportion of participants received ChAdOX1 (*n* = 28, 10.5%) and CoronaVac (*n* = 19, 7.1%).

### Distribution of SARS-CoV-2 antibody level (IgM, IgA, IgG, and NAb)

**Fig. 1:**
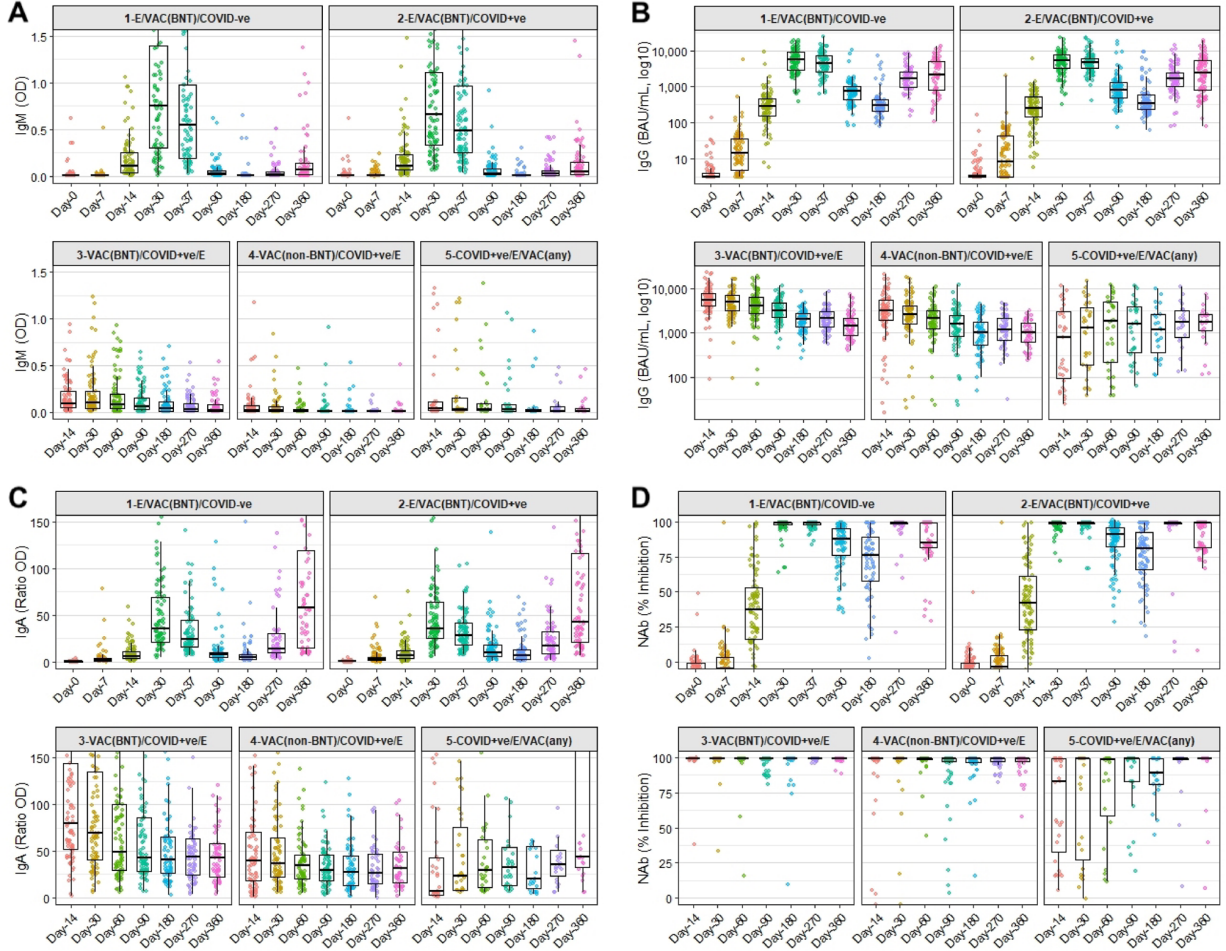
Kinetics of anti-SARS-CoV-2 S/RBD antibody levels in all timepoints over 12-months following BNT162b2 vaccination (top 2 graphs) and SARS-CoV-2 infection (bottom 3 graphs) in different study groups. (A) anti-RBD IgM, (B) anti-S IgG (log10 BAU/mL), (C) anti-S IgA, (D) anti-RBD neutralizing antibody. Antibody levels at each timepoint are shown as boxplots with the top and bottom sides of the box representing lower and upper quartiles, respectively, and the horizontal line splitting the box as the median level. The horizontal axis shows days from receipt of the first primary vaccine dose (top 2 graphs) or days from symptom onset of the most recent COVID-19 (bottom 3 graphs). *Abbreviations: SARS-CoV-2, Severe Acute Respiratory Syndrome Coronavirus 2; RBD, receptor-binding domain; IgM, immunoglobulin M; IgG, immunoglobulin G; IgA, immunoglobulin A; E, study enrollment; VAC(BNT), vaccinated with BNT162b2; VAC(non-BNT), vaccinated with a COVID-19 vaccine other than BNT162b2; VAC(any), vaccinated with any type of COVID-19 vaccine; COVID-ve, negative for COVID-19; COVID+ve, positive for COVID-19; "/" delineates sequential events in chronological order; ns, not significant.*

**Fig. 2:**
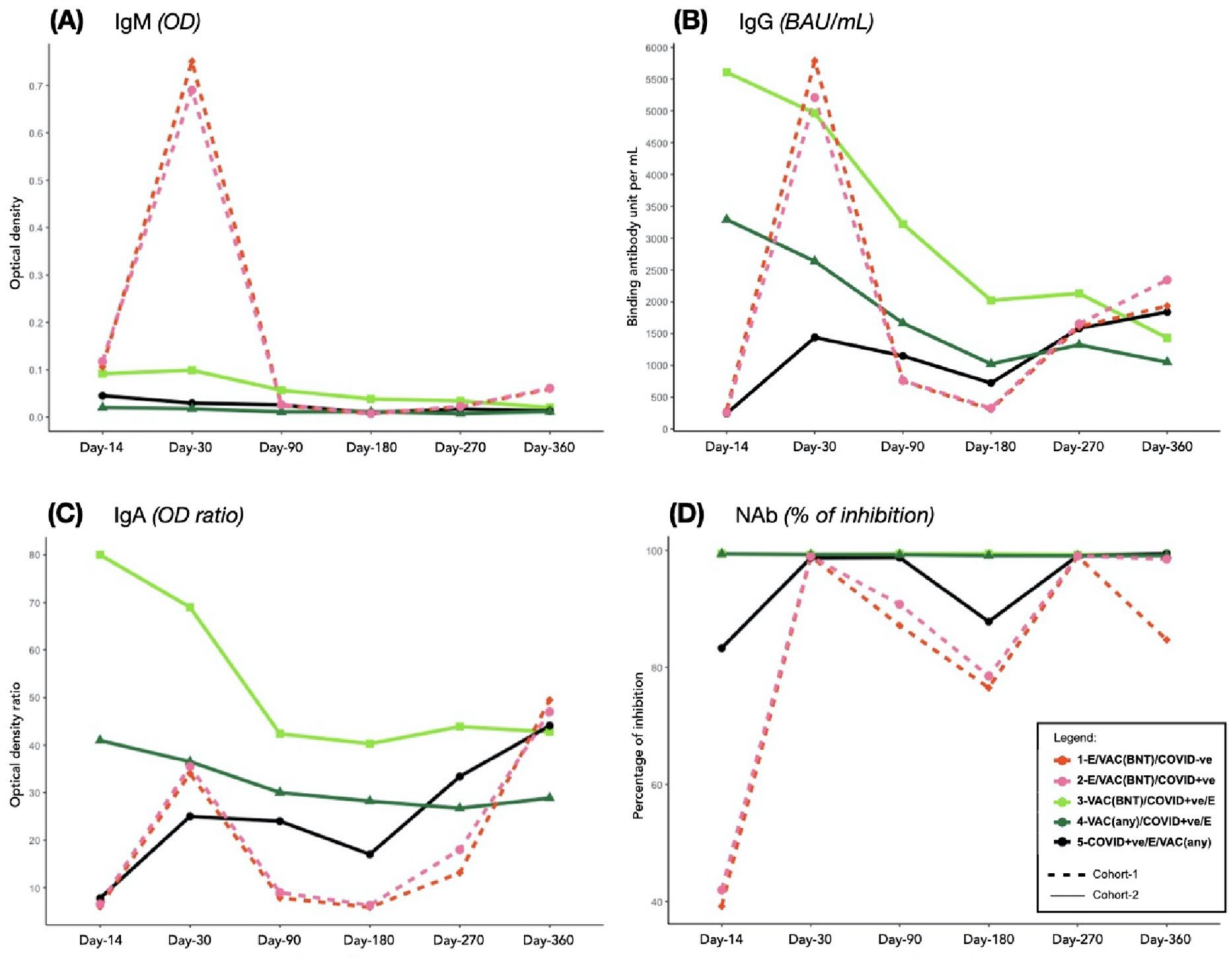
Longitudinal dynamics of SARS-CoV-2–specific antibody responses across study groups over a 12-month period. (A) Anti-SARS-CoV-2 IgM optical density (OD), (B) IgG binding antibody units (BAU/mL), (C) IgA OD ratio, and (D) neutralizing antibody capacity (% inhibition). For the 1-E/VAC(BNT)/COVID-ve and 2-E/VAC(BNT)/COVID+ve groups, timepoints are shown relative to the first primary BNT162b2 vaccination dose, whereas for the 3-VAC(BNT)/COVID+ve/E, 4-VAC(non-BNT)/COVID+ve/E, and 5-COVID+ve/E/VAC(any) groups, timepoints are shown relative to symptom onset of SARS-CoV-2 infection. Values represent median antibody levels for each study group. The legend in the lower right corner depicts the different study groups. *Abbreviations: SARS-CoV-2, Severe Acute Respiratory Syndrome Coronavirus 2; RBD, receptor-binding domain; IgM, immunoglobulin M; IgG, immunoglobulin G; IgA, immunoglobulin A; E, study enrollment; VAC(BNT), vaccinated with BNT162b2; VAC(non-BNT), vaccinated with a COVID-19 vaccine other than BNT162b2; VAC(any), vaccinated with any type of COVID-19 vaccine; COVID-ve, negative for COVID-19; COVID+ve, positive for COVID-19; "/" delineates sequential events in chronological order.*

Median positivity threshold levels of anti-SARS-CoV-2 RBD IgM were only observed in the 1-E/VAC(BNT)/COVID-ve and 2-E/VAC(BNT)/COVID+ve study groups (Fig. 1A). The IgM levels in these groups seroconverted to positivity on Day-14 (median 0.11 OD and 0.12 OD) and peaked on Day-30 (median 0.75 OD and 0.69 OD) (Fig. 2, Supplementary Fig. 2, Supplementary Table 1). However, the IgM seroreverted on Day-90 post-first vaccination dose and remained undetected subsequently.

The distribution of anti-SARS-CoV-2 S1 IgG, expressed as log10-transformed BAU/mL, exhibited a distinctive pattern across the five groups (Fig. 1B, Supplementary Fig. 2). IgG levels peaked on Day-30 in both 1-E/VAC(BNT)/COVID-ve and 2-E/VAC(BNT)/COVID+ve groups, then rapidly declined from Day-37. A gradual increase in IgG levels was observed on Day-270 and Day-360. However, seroconversion in these groups did not achieve the maximal IgG levels observed on Day-30 post-first BNT162b2 dose following re-exposure to SARS-CoV-2, either through subsequent infection or, in a subset of participants, receipt of a booster vaccination dose.

Following COVID-19, 3-VAC(BNT)/COVID+ve/E group exhibited a higher IgG levels compared to 4-VAC(non-BNT)/COVID+ve/E group, with a 52.0% difference at Day-14 and 61.2% at Day-30 post-symptom onset (Fig. 2). Nevertheless, the IgG levels declined rapidly in the 3-VAC(BNT)/COVID+ve/E group compared to the 4-VAC(non-BNT)/COVID+ve/E group during the first 180 days. Subsequently, the levels stabilized and became comparable from Day-180 to Day-360.

5-COVID+ve/E/VAC(any) group exhibited the lowest IgG level in comparison to groups that were vaccinated prior to infection. However, no significant seroconversion and seroreversion was observed. Moreover, this group exhibited a delayed IgG levels that gradually seroconverted from Day-14 to Day-30 in contrast to groups 3-VAC(BNT)/COVID+ve/E and 4-VAC(non-BNT)/COVID+ve/E, which had prior immunity from vaccination. However, the IgG levels in groups 3-VAC(BNT)/COVID+ve/E and 4-VAC(non-BNT)/COVID+ve/E, and 5-COVID+ve/E/VAC(any) remained stable and above the positivity threshold throughout all timepoints. IgG levels were closely clustered by Day-360 in all groups.

In groups 1-E/VAC(BNT)/COVID-ve and 2-E/VAC(BNT)/COVID+ve, anti-SARS-CoV-2 S IgA seroconverted on Day-30, seroreverted on Day-90, and remained stable on Day-180. However, there was a significant increase in IgA levels between Day-180 and Day-360 (Fig. 2C, Supplementary Fig. 2). IgA distribution pattern was comparable to that of IgG in groups 3-VAC(BNT)/COVID+ve/E and 4-VAC(non-BNT)/COVID+ve/E (Fig. 1B and C), which showed a peak after COVID-19 and stabilized at lower levels thereafter.

3-VAC(BNT)/COVID+ve/E group demonstrated the highest IgA levels on Day-14, which then decreased rapidly from Day-30 and reached a stable level by Day-90. 5-COVID+ve/E/VAC(any) group consistently exhibited elevated levels of IgA antibodies, surpassing those in 1-E/VAC(BNT)/COVID-ve and 2-E/VAC(BNT)/COVID+ve up until Day-270, with an increase from Day-180 to Day-360.

Neutralizing antibody (NAb) levels followed three patterns based on the primary exposure to SARS-CoV-2 among the study groups. These included naïve-SARS-CoV-2 BNT162b2 recipients (1-E/VAC(BNT)/COVID-ve and 2-E/VAC(BNT)/COVID+ve), vaccinated individuals with breakthrough infection (3-VAC(BNT)/COVID+ve/E and 4-VAC(non-BNT)/COVID+ve/E groups), and unvaccinated individuals prior to infection (5-COVID+ve/E/VAC(any) group) (Fig. 1D, Supplementary Fig. 2).

1-E/VAC(BNT)/COVID-ve and 2-E/VAC(BNT)/COVID+ve groups exhibited the lowest NAb percentage of inhibition (39.2% − 42%) on Day-14, which increased to 99% on Day-30 post-first BNT162b2 dose. The NAb percentage of inhibition waned from Day-90 to Day-180 and resurged to at least 99% on Day-270. By Day-360, the 1-E/VAC(BNT)/COVID-ve group showed a reduction in the percentage of inhibition, while the 2-E/VAC(BNT)/COVID+ve) group maintained a NAb percentage of inhibition of at least 98.5%.

Throughout the 360 days, both 3-VAC(BNT)/COVID+ve/E and 4-VAC(non-BNT)/COVID+ve/E consistently maintained the levels of NAb, demonstrating at least 99% inhibition. In the 5-COVID+ve/E/VAC(any) group, NAb levels increased from 83.3% to 98.7% between Day-14 and Day-30 following symptom onset and remained steady with at least 98% until Day-90. However, these levels declined on Day-180 but then recovered to at least 99%, and remained stable on Day-270 and Day-360.

### Correlation of antibody levels across sampling timepoints

**Fig. 3:**
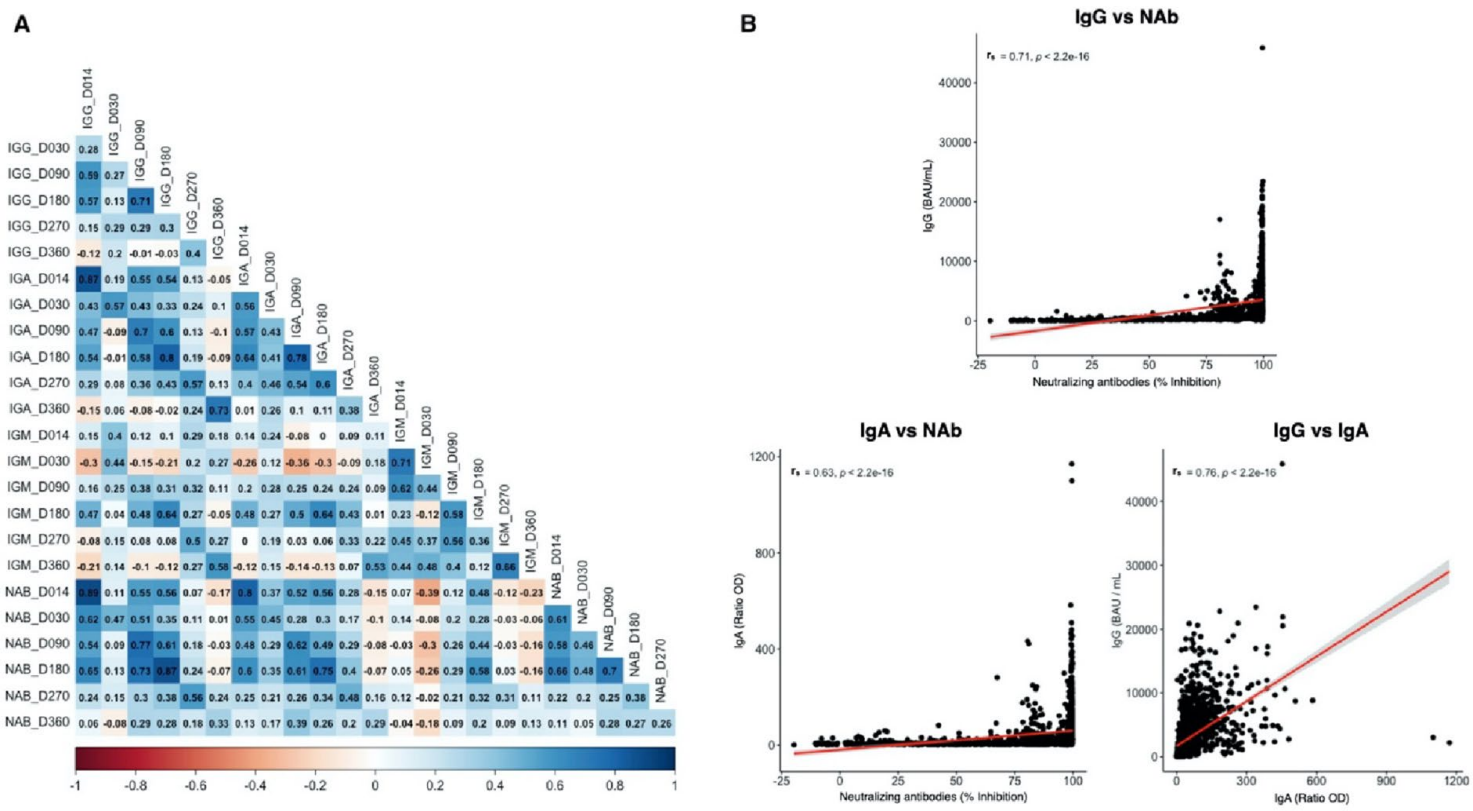
Correlation among antibody types and classes. (A) The heatmap illustrates the Spearman correlation coefficients of antibody levels between different antibody types (IgM, IgA, IgG, NAb) against the ancestral SARS-CoV-2 variant. The correlation matrix includes data from all sampling timepoints between days D014 to D360. The Spearman correlation coefficients between the different antibody types and sampling timepoints are color-coded as indicated in the figure. (B) Scatter plots with linear regression lines of SARS-CoV-2 neutralizing antibodies (NAb), IgG, and IgA; IgG vs. NAb, IgA vs. NAb, IgG vs. IgA. NAb, IgG, and IgA exhibited significant positive correlations with each other, with significance levels of *p* < 0.001. The highest correlation coefficient (r_s_) was observed between IgA and IgG, at 0.76 for moderate correlation between these two antibodies. The grey-shaded band areas represent the 95% confidence intervals. Correlations were assessed using Spearman’s rank correlation and therefore reflect relative associations between antibody responses rather than direct quantitative comparisons of absolute antibody levels across assays with different readouts and antigen targets.

Using Spearman’s rank correlation, we evaluated whether individuals with relatively higher antibody responses in one assay also tended to exhibit relatively higher responses in another, independent of assay scale or antigen format (Fig. 3). A strong positive correlation between NAb and IgG was observed on Day-14 and Day-180, with coefficient values of 0.89 and 0.87, respectively (Fig. 3 A). Similar correlation patterns were observed between NAb and IgA, however, did not reach statistical significance, with the highest correlation coefficient of 0.58 on Day-180.

Scatter plot analyses further demonstrated significant positive monotonic relationships between the levels of NAb, IgG and IgA (Fig. 3B). The Spearman correlation coefficient showed IgG exhibit a stronger positive correlation with NAb levels compared to IgA Fig. 3.

### Generalized estimating equation (GEE) analysis of antibody levels

**Fig. 4:**
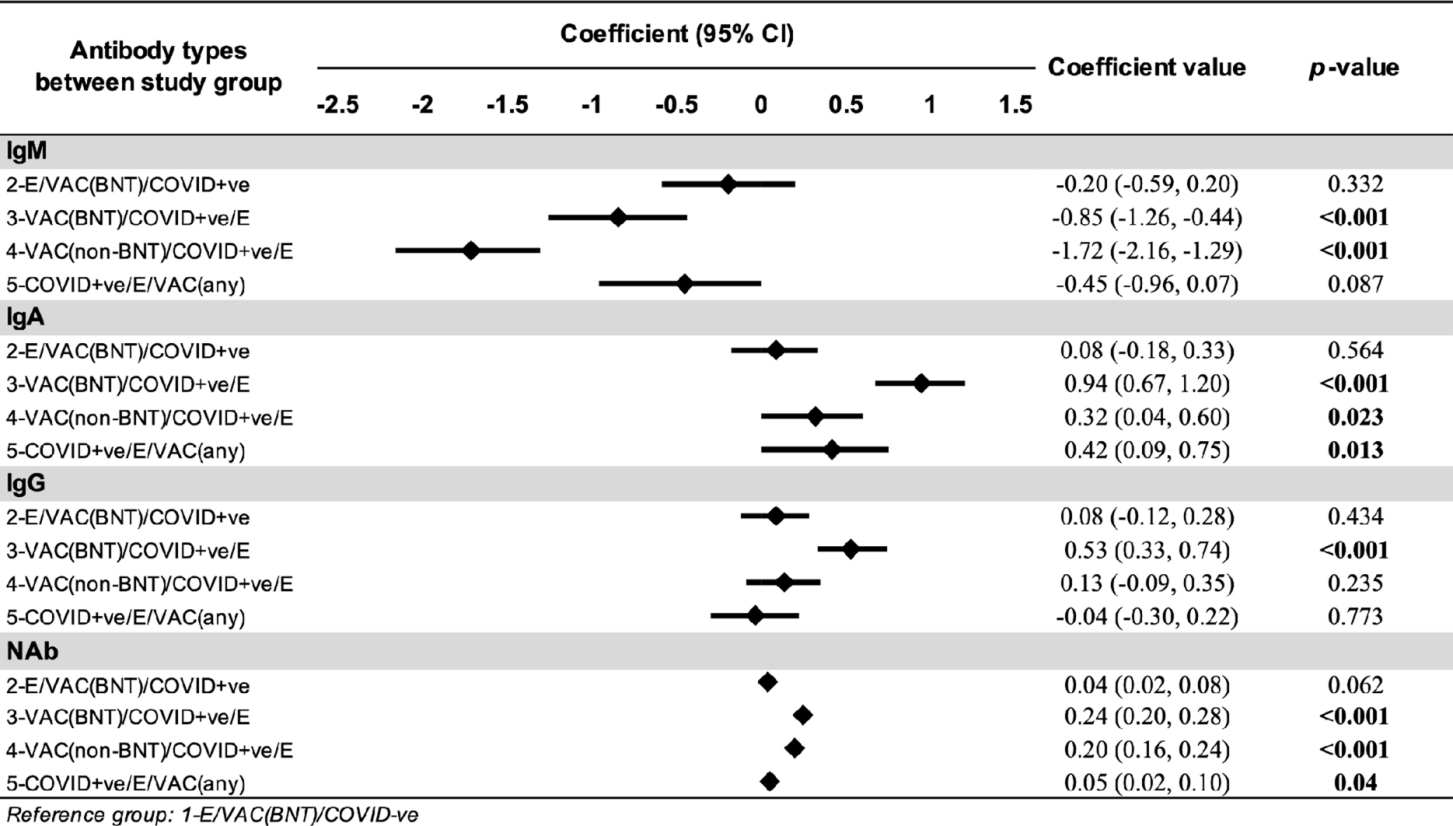
Forest plot represents the Generalized Estimating Equation (GEE) coefficients for temporal fluctuations in ancestral SARS-CoV-2 variant serum anti-RBD/anti-spike antibody level. These coefficients are shown across all sampling timepoints over the 12-month follow-up period, categorized by study group and antibody class. 1-E/VAC(BNT)/COVID-ve was set as the reference group as the participants in this group did not have a documented COVID-19 throughout the study period. Coefficients and *p*-values are shown for the estimates of antibody types between the study groups and the interaction between the longitudinal timepoints. The bolded *p*-values indicate a statistically significant effect of the changes in antibody levels during the study period.

The 1-E/VAC(BNT)/COVID-ve group was set as the reference group for Generalized Estimating Equation (GEE) models (Fig. 4). 3-VAC(BNT)/COVID+ve/E and 4-VAC(non-BNT)/COVID+ve/E groups consistently exhibited significantly lower IgM levels compared to 1-E/VAC(BNT)/COVID-ve. Specifically, in group 3-VAC(BNT)/COVID+ve/E, IgM levels were 0.85 units lower (95% CI: −1.26, −0.44; *p* < 0.001), whereas 4-VAC(non-BNT)/COVID+ve/E exhibited a greater lower of −1.72 units (95% CI: −2.16, −1.29; *p* < 0.001) relative to 1-E/VAC(BNT)/COVID-ve.

3-VAC(BNT)/COVID+ve/E, 4-VAC(non-BNT)/COVID+ve/E, and 5-COVID+ve/E/VAC(any) groups exhibited significantly higher IgA levels than 1-E/VAC(BNT)/COVID-ve, with values of 0.94 units (95% CI: 0.67, 1.20), 0.32 units (95% CI: 0.04, 0.60), and 0.42 units (95% CI: 0.09, 0.75), respectively. Additionally, the IgG levels in 3-VAC(BNT)/COVID+ve/E were significantly higher by 0.53 units (95% CI: 0.33, 0.74) than those in 1-E/VAC(BNT)/COVID-ve.

The NAb levels in 3-VAC(BNT)/COVID+ve/E and 4-VAC(non-BNT)/COVID+ve/E groups were significantly higher than those of 1-E/VAC(BNT)/COVID-ve by 0.24 (95% CI: 0.20, 0.28) and 0.20 (95% CI: 0.16, 0.24), respectively. 5-COVID+ve/E/VAC(any) group exhibited a minimal difference in NAb levels compared to the reference group, with a GEE coefficient of 0.05 (95% CI: 0.002, 0.10), despite a significant change in NAb levels over time.

### Incidence and time to COVID-19 following primary vaccination and prior infection

COVID-19 incidence rates varied across the study groups. The analysis examined the time-to-infection following the first and second primary vaccination doses, as well as ‘complete vaccination’ status, which was defined as at least 14 days after the second dose (referred to as “Day-15 after the second dose”).

**Fig. 5:**
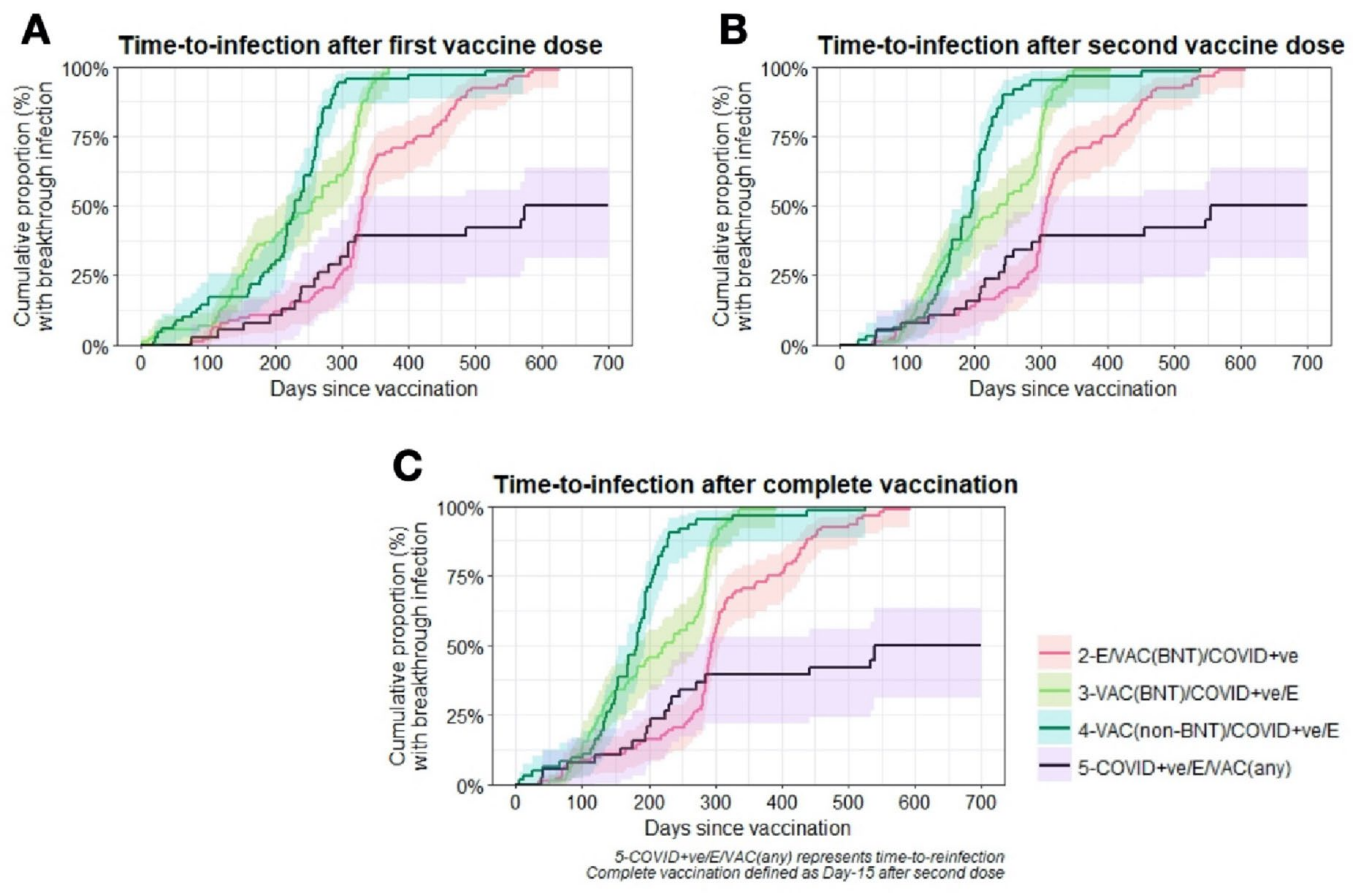
Kaplan-Meier curves show the cumulative probability of acquiring a SARS-CoV-2 infection over time in distinct post-vaccination periods, stratified by study groups. (A) Time to infection since the first primary series vaccination dose (B) Time to infection since the second primary series vaccination and (C) Time to infection since Day-15 after the second primary vaccine series dose. The lines show the proportion of the participants with documented breakthrough COVID-19 following vaccination; the color-shaded indicates the pointwise 95% confidence intervals.

Across all post-vaccination phases, the 3-VAC(BNT)/COVID+ve/E and 4-VAC(non-BNT)/COVID+ve/E groups consistently exhibited the highest incidences of COVID-19 cases (Fig. 5 and Supplementary Table 2). However, the Kaplan-Meier logrank test analysis showed no significant differences between these groups (*p* = 0.065) (Supplementary Table 3). The 2-E/VAC(BNT)/COVID+ve group exhibited slightly lower rates of COVID-19 ranging from − 0.03 to −1.4 compared to other breakthrough infection groups, which were 3-VAC(BNT)/COVID+ve/E and 4-VAC(non-BNT)/COVID+ve/E.

5-COVID+ve/E/VAC(any) consistently exhibited the lowest incidence rate of COVID-19 re-infection over the 12-month study period, irrespective of the vaccination phases. Analysis of time to second episode of COVID-19 following the completion of primary vaccination demonstrated a significantly prolonged infection-free interval in this group compared with all other study groups (*p* < 0.001) (Supplementary Table 3). Overall, COVID-19 incidence rates showed an inverse relationship with the median time to COVID-19 across vaccination phases (*p* < 0.01) (Fig. 5 and Supplementary Table 2).

### Incidence and time-to-breakthrough infection by antibody level (Low, Moderate, High)

A comparative analysis of breakthrough infection (BTI) incidence across antibody responder categories (Low, Moderate, and High) was performed to explore potential trends during complete vaccination (Day-37) and the early antibody waning period (Day-90) (Supplementary Table 4). While moderate IgG responders exhibited the highest BTI incidence rate at Day-37 and high NAb responders at Day-90, the confidence intervals overlapped across all responder categories. Consistent with this, no statistically significant differences in BTI incidence rates were observed between antibody responder groups at either timepoint. These findings indicate that stratification by antibody level alone did not clearly discriminate BTI risk during these periods.

### Comparison of IgG levels across exposures, waning, and 12-month follow up

**Fig. 6:**
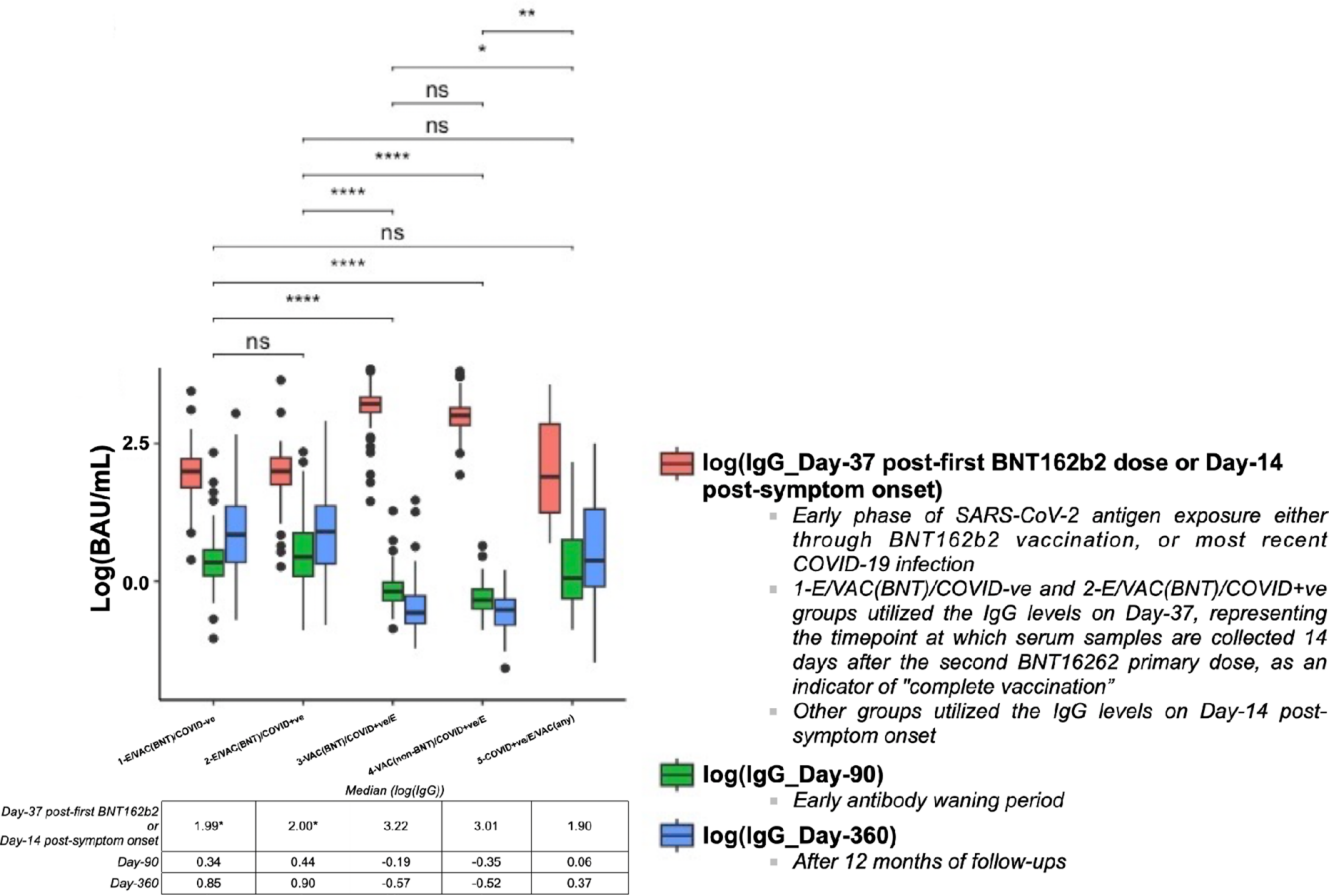
The rate changes of SARS-CoV-2 serum anti-S1 median log-IgG levels in early period, Day-90, and Day-360 of SARS-CoV-2 antigen exposure across different study groups. 5-COVID+ve/E/VAC(any) group showed a more sustained IgG levels compared to those who were vaccinated without prior infection. Median log-IgG levels are shown below each study group and timepoint. Mann-Whitney U test was performed to measure the magnitude of the rate changes in log-IgG levels between the study groups. Significant differences are marked as *p < 0.05, **p < 0.01, ***p < 0.001, ****p < 0.0001. No significant differences were marked in plot where with “ns” (not significant) is indicated. Abbreviations: SARS-CoV-2, Severe Acute Respiratory Syndrome Coronavirus 2; IgG, immunoglobulin G; E, study enrollment; VAC(BNT), vaccinated with BNT162b2; VAC(non-BNT), vaccinated with a COVID-19 vaccine other than BNT162b2; VAC(any), vaccinated with any type of COVID-19 vaccine; COVID-ve, negative for COVID-19; COVID+ve, positive for COVID-19; “/“ delineates sequential events in chronological order.

To assess antibody durability across exposure groups, median log-IgG levels were compared at three predefined immunologically relevant timepoints: (i) Early response post-SARS-CoV-2 antigen exposure (where 1-E/VAC(BNT)/COVID-ve and 2-E/VAC(BNT)/COVID+ve utilized median log-IgG levels on Day-37 post-first BNT162b2 vaccination dose which represents the “complete BNT162b2 primary vaccination doses”, and other groups utilized median log-IgG levels on Day-14 post-symptom onset which represents “complete antibody levels following infection”), (ii) Day-90 (early antibody waning period), and (iii) Day-360 (after 12-month follow-up) (Fig. 6).

No significant differences in median log-IgG proportion between the 1-E/VAC(BNT)/COVID-ve and 2-E/VAC(BNT)/COVID+ve groups, as well as between the 3-VAC(BNT)/COVID+ve/E and 4-VAC(non-BNT)/COVID+ve/E groups. The rate changes in 5-COVID+ve/E/VAC(any) were comparable to those in 1-E/VAC(BNT)/COVID-ve and 2-E/VAC(BNT)/COVID+ve. The baseline median log-IgG levels were significantly lower in the 5-COVID+ve/E/VAC(any) group (*p* < 0.01) compared to the 3-VAC(BNT)/COVID+ve/E and 4-VAC(non-BNT)/COVID+ve/E groups. However, by Day-360, the median log-IgG levels in the 5-COVID+ve/E/VAC(any) group were significantly higher (*p* < 0.01) than in the other two groups.

### Comparison of IgG levels to different vaccine platforms following a breakthrough infection

**Fig. 7:**
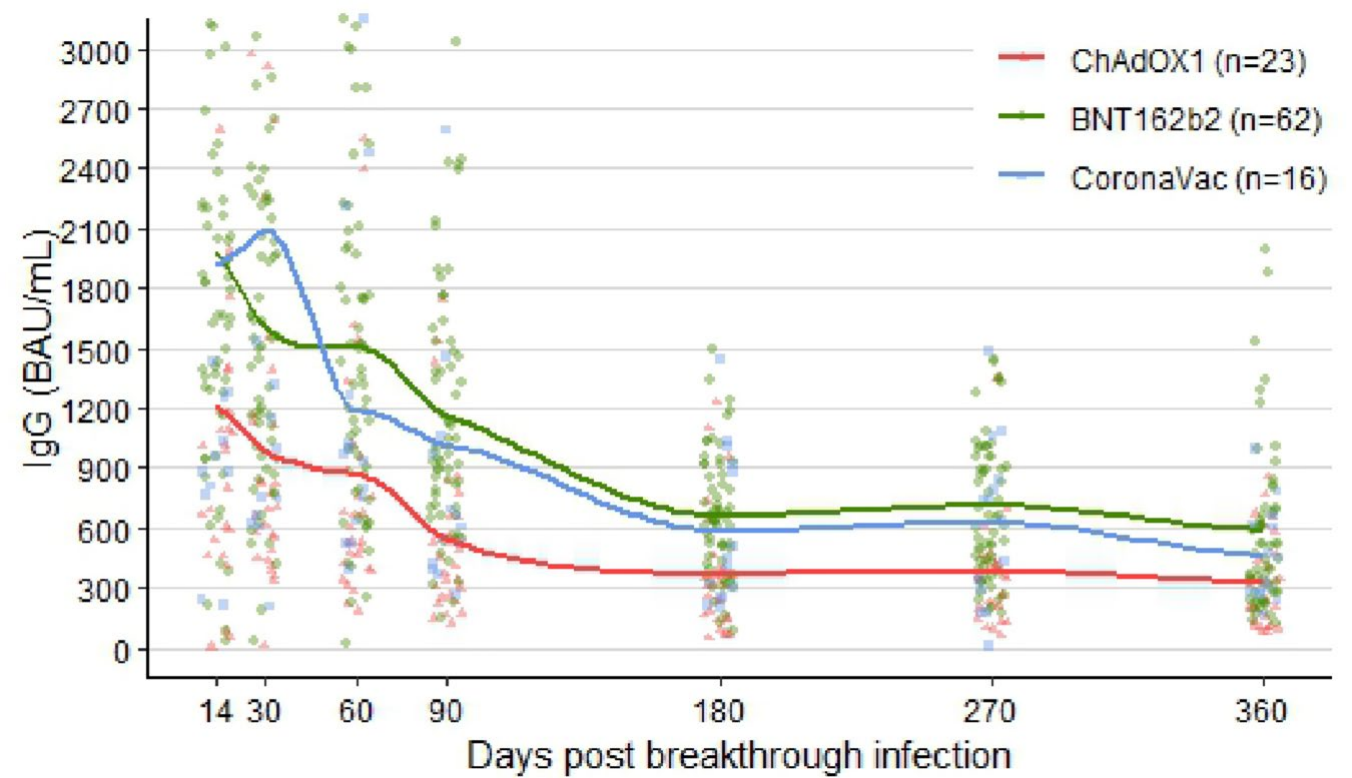
Longitudinal distribution of IgG levels (BAU/mL) across different COVID-19 vaccination types. Stratification of the data is based on the ChAdOX1 (*n* = 23), BNT162b2 (*n* = 62), and CoronaVac (*n* = 16) vaccines administered. Regression lines were performed among the vaccines using the median IgG level and the 95% confidence interval of the regression line is denoted by the grey color.

3-VAC(BNT)/COVID+ve/E and 4-VAC(non-BNT)/COVID+ve/E groups demonstrated a wide range of IgG levels until Day-60 or for up to the first two months following breakthrough infections (BTI). At later timepoints, the IgG levels nearly converged towards a similar plateau despite the initial heterogeneity, irrespective of the vaccine types (Fig. 7).

### Comparison of IgG levels by age group and sex across vaccination phases

**Fig. 8:**
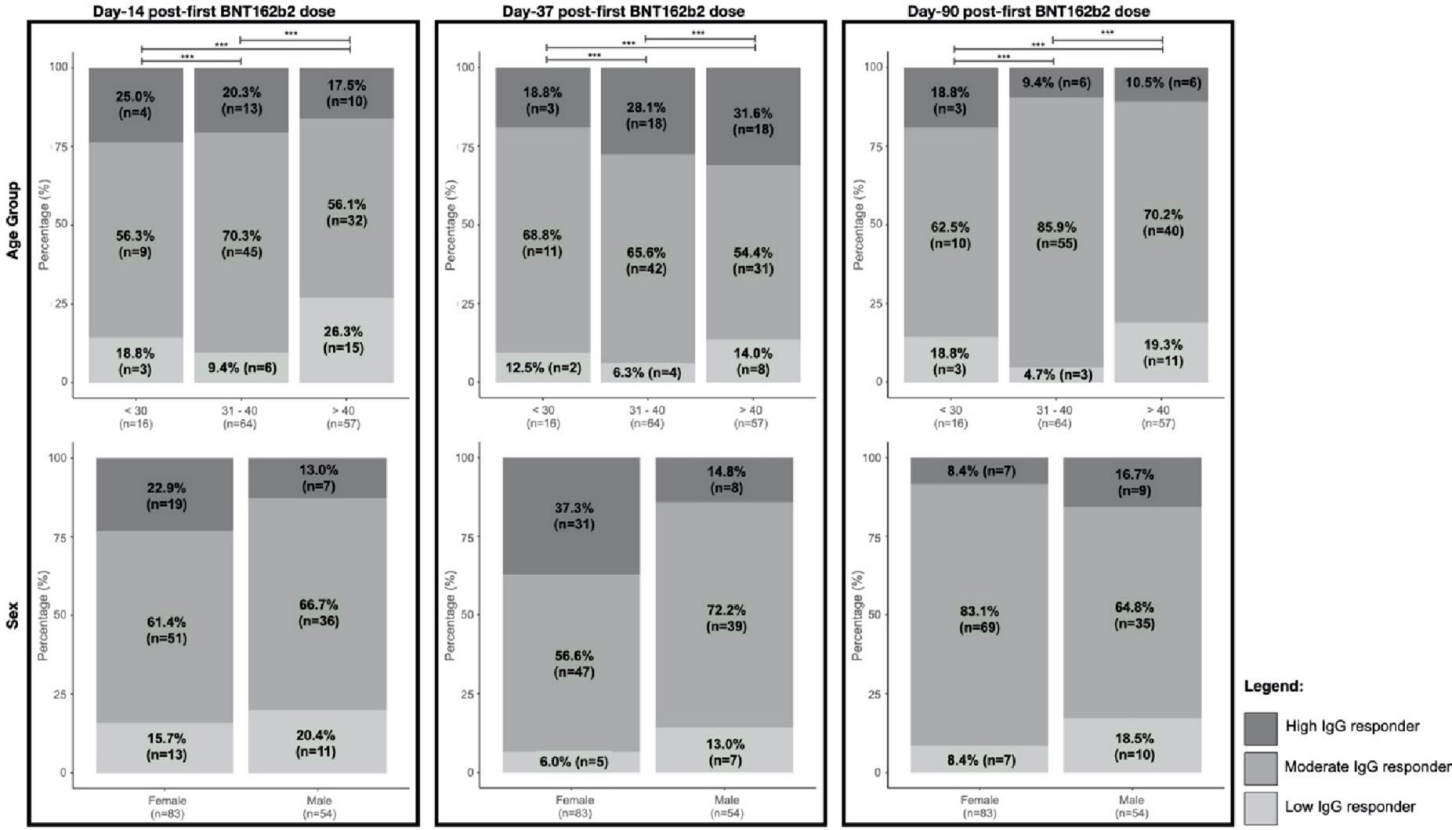
Distribution of IgG level post-vaccination according to age group and sex. The IgG responders were categorized into low (< 25th percentile), moderate (25th − 75th percentile), and high (> 75th percentile) among participants (*n* = 137). A significant difference in antibody level categories was observed across age groups (*p* < 0.001), while no significant difference was found between sexes, as determined by the Chi-square test. Significant differences are marked as *p < 0.05, **p < 0.01, ***p < 0.001, ****p < 0.0001.

The determination of low, high, and moderate IgG levels of 1-E/VAC(BNT)/COVID-ve and 2-E/VAC(BNT)/COVID+ve group (*n* = 137) were based on their percentiles. There was a statistically significant difference in IgG levels categories across different age groups (*p* < 0.001). On Day-14 post-first BNT162b2 dose (primary vaccination response period), participants under the age of 30 exhibited the highest proportion of high IgG responders (25.0%). By Day-37 post-first BNT162b2 dose (complete vaccination period), participants over the age of 40 accounted for the majority of high IgG responders (31.6%). However, by Day-90 post-first BNT162b2 dose (early waning antibody period), the proportion pattern resembled the primary vaccination response period, with participants under 30 showing the highest IgG levels category (18.8%) (Fig. 8).

Female participants exhibited a higher proportion of high IgG responders in Day-14 and Day-37 post-first BNT162b2 dose (*n* = 19, 22.9%) and the complete vaccination period (*n* = 31, 37.3%) compared to male participants (*n* = 7, 13.0%; *n* = 8, 14.8%), respectively. In the Day-90 post-first BNT162b2 dose, more males (*n* = 9, 16.7%) exhibited high IgG levels compared to females (*n* = 7, 8.4%), with most females demonstrating a moderate level (*n* = 69, 83.1%). These differences were not statistically significant (Fig. 8).

## Discussion

Understanding long-term SARS-CoV-2 antibody levels is essential for assessing humoral immunity, as neutralizing antibodies (NAb) correlate with protection against symptomatic infection^11,15–17^. In contrast to prior studies that focus on vaccine-specific responses or short-term antibody kinetics, the present study integrates long-term serological trajectories with breakthrough and reinfection outcomes across distinct exposure sequences. We investigated the dynamics and durability of serum IgM, IgA, IgG, and NAb against the receptor-binding-domain (RBD) or spike (S) protein of ancestral SARS-CoV-2 variant in five study groups with varying SARS-CoV-2 exposures over 12-months, assessing their relation to breakthrough infections (BTI) or reinfection.

Group-specific analysis revealed that primary BNT162b2 vaccination significantly elevated IgA, IgG, and NAb levels by 7 days following the second dose (Day-30 in our sampling timepoint). No significant increase in level after the first dose, emphasizing the necessity for a minimum of two BNT162b2 doses to achieve sufficient protection against the ancestral SARS-CoV-2 variant. However, antibody levels waned over the subsequent 7 days, reaching a lower level 14 days after the second dose (Day-37 in our sampling timepoint). Previous studies of vaccine efficacy showed a similar waning trend in anti-S antibody levels approximately 21 to 41 days after the second dose of BNT162b2 and ChAdOX1 in SARS-CoV-2-naïve individuals^14^. Antibody distribution patterns in our study indicated a rapid decline in antibody levels weeks following primary vaccination, although the decrease was not statistically significant. Our findings were consistent with other observed declines in antibody and NAb levels against the spike protein following SARS-CoV-2 infection^9,15^. The waning of antibodies in the early phase of mass vaccination raises concerns over the long-term efficacy and durability of the primary doses of BNT162b2 vaccine even against the ancestral SARS-CoV-2 variant lineage^11,16^.

Serum IgA, IgG, and NAb levels in BNT162b2-vaccinated groups declined by Day-90 post-first dose, reaching the lowest levels by Day-180 post-first dose. Consistent with Kaplan-Meier time-to-infection analysis, waning antibody level from the primary-two doses of BNT162b2 vaccination was associated with increased breakthrough infection and reinfection^17^, especially with emerging Omicron lineages and sub-lineages^8^. Booster doses administered between Day-270 and Day-360 significantly restored antibody levels, reducing symptomatic infection^3,18^. Boosters also enhanced hybrid immunity in previously infected individuals, sustaining higher antibody levels and conferring stronger protection against reinfection^3^.

We observed no significant difference in breakthrough infections (BTI) rates between vaccine platforms (BNT162b2 vs. non-BNT162b2 vaccines such as ChAdOx1, CoronaVac). However, BNT162b2-vaccinated individuals with BTI exhibited higher IgA, IgG, and NAb levels over 12-month, likely reflecting repeated antigen exposure following infection. Vaccine effectiveness in preventing BTI and reinfection may depend on humoral and cellular immunity^10^. While BTI occurred in both 2-E/VAC(BNT)/COVID+ve and 3-VAC(BNT)/COVID+ve/E groups, their antibody trajectories differed due to initial SARS-CoV-2 exposure. Over time, antibody levels in the 2-E/VAC(BNT)/COVID+ve group may align with 3-VAC(BNT)/COVID+ve/E. Notably, BTI cases across all vaccine platforms exhibited high RBD-ACE2-blocking neutralizing capacity (> 90%) throughout, indicating robust and sustained humoral immune responses^7^. In addition, six months post-infection, our study showed that the IgG levels were comparable across vaccine types, suggesting similar BTI risk regardless of primary vaccination.

The persistence of IgA, IgG, and NAb levels in group with SARS-CoV-2 infection prior to vaccination (5-COVID+ve/E/VAC(any)) is likely due to the memory B cell formation during the interval between infection and subsequent vaccinations^7^. This group also had the lowest incidence of SARS-CoV-2 re-infection, suggesting that hybrid immunity from previous infection before vaccination induces long-lasting protection compared with vaccination in infection-naïve individuals^19^. Our Kaplan-Meier analysis further demonstrated that participants with prior SARS-CoV-2 infection before vaccination had the lowest probability of reinfections, with 50% remained free from documented reinfection for up to 700 days post- primary vaccine. The protective effect of hybrid immunity observed in our study aligns with previous findings^18,20,21^. Notably, individuals who had recovered from COVID-19 exhibited significantly higher and more durable antibody level following a single dose of BNT162b2, mRNA-1273, or ChAdOX1^20^. This enhanced response was even more effectiveness in reducing reinfection risk after completing two vaccine doses^22^. However, during the Delta variant surge, no significant difference in reinfection risk was observed between those who received one or two doses of BNT162b2 after prior COVID-19^23^. These findings suggest that a single-dose vaccination strategy may be a viable approach for individuals with confirmed COVID-19, particularly in areas with limited vaccine availability.

Consistent with other studies, our study observed a stronger correlation between IgG and NAb than between IgA and NAb, suggesting that neutralizing activity in this cohort was primarily mediated by IgG. This finding aligns with previous studies identifying IgG as the main immunoglobulin mediating protective response against SARS-CoV-2 infections^24,25^. Further assessment of IgG levels by sex and age revealed that age plays a more significant role than sex in determining the antibody levels and their durability. Notably, antibody levels followed a biphasic trend across different age groups. While elderly participants generally exhibited lower antibody levels than those under 40 during the early and waning phases post-vaccination, they displayed the highest antibody levels at peak response. This suggests a non-linear relationship between age and IgG levels throughout the peri-vaccination period. Lower antibody levels in older individuals may be attributed to immunosenescence, including reduced germinal center size and function^6^. Studies on booster vaccinations in the elderly have yielded diverse results, with some reporting impaired T cell responses post-booster^26^, while others indicate improved immunity^27^. Nonetheless, booster immunization remains beneficial in reducing COVID-19 risk among older individuals^28^, emphasizing the need for routine immunity screening in this population.

A key strength of our study is its prospective longitudinal cohort design, spanning 12-month with a high retention rate (66.8%, *N* = 267/400). While some participants were unable to complete sampling due to collection occurring during different phases of the local movement control order^13^, this design allowed us to differentiate participants based on their SARS-CoV-2 exposure histories. Additionally, our sampling period spanned from the early rollout of COVID-19 primary vaccination to three years of the pandemic (i.e., from March 2021 to May 2023), encompassed the Delta, pre-Omicron, and post-Omicron variant predominance periods. Another strength is our comprehensive monitoring of all four primary immunoglobulin classes (IgM, IgA, IgG, and NAb) against the S protein or RBD at up to nine timepoints. Unlike other antibody classes, IgM is less frequently characterized in SARS-CoV-2 studies, as it is primarily used for diagnostic purposes. However, we tracked the IgM levels due to its reported higher virus-neutralizing capacity against lentiviral particles pseudotyped with SARS-CoV-2 S protein, as demonstrated in previous studies^29^.

There are several limitations in this study. First, a decline in antibody level over time is a natural aspect of humoral immunity. While NAb levels serve as a strong indicator of protection, antibody kinetics vary across exposure groups. To address this, we plan to evaluate the cellular responses in selected participants using previously collected peripheral blood mononuclear cells. Second, our study focused exclusively on antibodies targeting the ancestral SARS-CoV-2 variant, which is no longer predominant. As the study period spanned Delta- and Omicron-dominant waves, most breakthrough infections likely involved non-ancestral variants. While binding antibody responses are generally more cross-reactive across variants, neutralizing antibody responses, particularly those measured by RBD-ACE2 competition, are more sensitive to antigenic variation. Furthermore, the surrogate neutralization assay used in this study quantifies antibodies that inhibit RBD-ACE2 binding and therefore primarily captures neutralizing responses directed toward epitopes overlapping the receptor-binding domain. Neutralizing antibodies targeting other regions of the spike protein, including non-RBD epitopes, may not be detected and could therefore underestimate the breadth of functional humoral immunity. Accordingly, variant-specific neutralization assays would provide additional resolution in defining protection against circulating SARS-CoV-2 variants in future studies. Third, immune responses were aggregated to facilitate population level comparisons, which may have masked individual immune variations. Lastly, reliance on documented symptomatic infection episodes and the relatively long intervals between sampling timepoints have led to an underestimation of BTI and reinfection incidence. Future studies incorporating more frequent sampling and respiratory specimen collection would improve detection sensitivity.

## Conclusion

In summary, our study revealed distinct longitudinal antibody profiles across different SARS-CoV-2 exposure histories, including vaccination alone, infection, or hybrid immunity. Antibody level following BNT162b2 vaccination exhibited a biphasic pattern, characterized by an initial rapid decline within 14 days post-second dose, followed by a stabilization phase lasting until Day 360. After a third exposure, either from a breakthrough infection or booster vaccination, antibody kinetics differed. Specifically, antibody levels remained elevated in previously infected individuals. Whereas boosted individuals without prior infection exhibited a waning trend similar to those observed after primary vaccination. Despite higher antibody levels among BNT162b2, breakthrough infection risk and time-to-infection were comparable across vaccine platforms. Notably, hybrid immunity, resulting from SARS-CoV-2 infection prior to vaccination was associated with prolonged antibody levels and reduced reinfection risk. These findings highlight the importance of future SARS-CoV-2 vaccine strategies that elicit durable anti-spike immune responses across diverse exposure histories, with variant-specific immunity requiring further dedicated investigation. Together, these findings suggest that exposure history may be an important consideration alongside vaccine platform when evaluating long-term population immunity against SARS-CoV-2.

## Supplementary Information

Below is the link to the electronic supplementary material.

Supplementary Material 1

## Data Availability

The datasets generated during and/or analyzed in this study are available from the corresponding authors on reasonable request.
