# Supplementary material for: Hybrid immunity following SARS-CoV-2 infection and vaccination is associated with more sustained antibody levels, and lower reinfection risk
Source: Sci Rep. 2026 May 29;16:24595. doi: 10.1038/s41598-026-55396-x (PMC13454461; doi:10.1038/s41598-026-55396-x)
Supplement: Supplementary file 1 [file 41598_2026_55396_MOESM1_ESM.docx]

**Supplementary**

**Supplementary Figure 1**. Schematic representation of the serum collection timepoints.

*Abbreviations: E, study enrollment; VAC(BNT), vaccinated with BNT162b2; VAC(non-BNT), vaccinated with a COVID-19 vaccine other than BNT162b2; VAC(any), vaccinated with any type of COVID-19 vaccine; COVID-ve, negative for COVID-19; COVID+ve, positive for COVID-19; "/" delineates sequential events in chronological order.*

**Supplementary Figure 2**. Kinetics of anti-SARS-CoV-2 S/RBD antibody levels in all timepoints over 12-months following BNT162b2 vaccination (top 2 graphs) and SARS-CoV-2 infection (bottom 3 graphs) in different study groups. (A) anti-RBD IgM, (B) anti-S IgG (log10 BAU/mL), (C) anti-S IgA, (D) anti-RBD neutralizing antibody. Antibody levels at each timepoint are shown as boxplots with the top and bottom sides of the box representing lower and upper quartiles, respectively, and the horizontal line splitting the box as the median level. The horizontal axis shows days from receipt of the first primary vaccine dose (top 2 graphs) or days from symptom onset of the most recent COVID-19 (bottom 3 graphs). Mann-Whitney U test was performed to show median differences between the timepoints within each group. Significant differences are marked as **p* < 0.05, ***p* < 0.01, ****p* < 0.001, *****p* < 0.0001. In plots where only "ns" is displayed, the differences between other timepoints are significant and vice versa.

*Abbreviations: SARS-CoV-2, Severe Acute Respiratory Syndrome Coronavirus 2; RBD, receptor-binding domain; IgM, immunoglobulin M; IgG, immunoglobulin G; IgA, immunoglobulin A; E, study enrollment; VAC(BNT), vaccinated with BNT162b2; VAC(non-BNT), vaccinated with a COVID-19 vaccine other than BNT162b2; VAC(any), vaccinated with any type of COVID-19 vaccine; COVID-ve, negative for COVID-19; COVID+ve, positive for COVID-19; "/" delineates sequential events in chronological order; ns, not significant.*

**Supplementary Table 1.** Quantitative SARS-CoV-2 anti-RBD/anti-S antibody levels across sampling timepoints during the 12-month follow up period by study groups.

*Abbreviations: SARS-CoV-2, Severe Acute Respiratory Syndrome Coronavirus 2; RBD, receptor-binding-domain; S, spike protein, E, study enrollment; VAC(BNT), vaccinated with BNT162b2; VAC(non-BNT), vaccinated with a COVID-19 vaccine other than BNT162b2; VAC(any), vaccinated with any type of COVID-19 vaccine; COVID-ve, negative for COVID-19; COVID+ve, positive for COVID-19; "/" delineates sequential events in chronological order.*

| **Study group** | **Day post-exposure** | | | | | |
| --- | --- | --- | --- | --- | --- | --- |
|  | **14** | **30** | **90** | **180** | **270** | **360** |
|  | Median  (Min-Max) | Median  (Min-Max) | Median  (Min-Max) | Median  (Min-Max) | Median  (Min-Max) | Median  (Min-Max) |
| **IgM** *(OD, Cut off value = 0.105)* | | | | | | |
| 1-E/VAC(BNT)/COVID-ve | 0.11  (0.01-1.06) | 0.75  (0.04-3.55) | 0.02  (0.01-0.62) | 0.01  (0.01-0.41) | 0.02  (0.01-0.51) | 0.06  (0.01-0.11) |
| 2-E/VAC(BNT)/COVID+ve | 0.12  (0.01-1.79) | 0.69  (0.05-3.58) | 0.03  (0.01-0.92) | 0.01  (0.01-0.41) | 0.02  (0.01-0.51) | 0.06  (0.01-0.12) |
| 3-VAC(BNT)/COVID+ve/E | 0.09  (0.01-0.94) | 0.1  (0.01-1.23) | 0.06  (0.01-0.64) | 0.04  (0.01-0.71) | 0.03  (0.01-0.53) | 0.02  (0.01-0.09) |
| 4-VAC(non-BNT)/COVID+ve/E | 0.02  (0.01-2.74) | 0.02  (0.01-0.84) | 0.01  (0.01-0.51) | 0.01  (0.01-0.53) | 0.01  (0.01-0.12) | 0.01  (0.01-0.02) |
| 5-COVID+ve/E/VAC(any) | 0.05  (0.01-3.5) | 0.03  (0.01-2.7) | 0.03  (0.01-1.07) | 0.01  (0.01-0.87) | 0.02  (0.01-0.48) | 0.01  (0.01-0.05) |
| **IgA** *(OD ratio, Cut off value =1.1)* | | | | | | |
| 1-E/VAC(BNT)/COVID-ve | 6.1  (0.7-59) | 34  (5-155) | 7.9  (1-465) | 5.9  (1.4-150) | 13.2  (3.6-203) | 49.5  (8-433) |
| 2-E/VAC(BNT)/COVID+ve | 6.6  (0.4-281) | 35.5  (6-583) | 9  (0.6-139) | 6.4  (1.1-69.7) | 18  (2-207) | 47  (7-422) |
| 3-VAC(BNT)/COVID+ve/E | 80  (1.6-388) | 69  (6-327) | 42.4  (7-372) | 40.3  (3.2-1170) | 43.9  (3.8-1100) | 42.8  (4.3-412) |
| 4-VAC(non-BNT)/COVID+ve/E | 41  (1-339) | 36.5  (6-390) | 30  (3-124) | 28.2  (1.6-128) | 26.7  (0-334) | 28.9  (2.4-481) |
| 5-COVID+ve/E/VAC(any) | 7.8  (0-153) | 25  (6-146) | 24  (7-387) | 17.1  (2-278) | 33.4  (4.4-247) | 44.1  (6-266) |
| **IgG** *(BAU/mL, Cut off value =35.2)* | | | | | | |
| 1-E/VAC(BNT)/COVID-ve | 296.32 (5.76-4156.8) | 5788.8  (379.52-19241.6) | 752.64  (82.56-10636.8) | 307.84  (78.4-7008) | 1608  (151.68-7910.4) | 1934.4  (109.12-11411.2) |
| 2-E/VAC(BNT)/COVID+ve | 256.96 (5.76-9155.2) | 5208  (320-45856) | 761.6  (74.56-18400) | 328  (63.04-9337.6) | 1652.48  (78.72-14035.2) | 2343.04  (77.76-18953.6) |
| 3-VAC(BNT)/COVID+ve/E | 5606.4 (90.88-23427.2) | 4963.2  (94.72-17011.2) | 3222.4  (464-11734.4) | 2021.12  (267.52-8883.2) | 2130.56  (566.72-8748.8) | 1431.36  (387.84-7462.4) |
| 4-VAC(non-BNT)/COVID+ve/E | 3292.48 (25.6-20857.6) | 2636.8  (19.84-18828.8) | 1664  (23.04-12051.2) | 1023.04  (49.6-4617.6) | 1323.2  (32-4758.4) | 1050.56  (244.16-3180.48) |
| 5-COVID+ve/E/VAC(any) | 244.48 (15.68-12022.4) | 1440  (30.08-14729.6) | 1148.8  (63.04-12003.2) | 723.2  (106.88-10256) | 1582.72  (132.16-11340.8) | 1838.08  (112-9206.4) |
| **NAb** *(% of inhibition)* | | | | | | |
| 1-E/VAC(BNT)/COVID-ve | 39.2  (-19.7-97.6) | 99  (64-99.5) | 87.1  (37.1-99.5) | 76.5  (2.3-99.5) | 99  (21-99.5) | 84.7  (29.4-99.6) |
| 2-E/VAC(BNT)/COVID+ve | 42  (-10.4-99.4) | 99  (67.1-99.6) | 90.8  (28.5-101.5) | 78.5  (15.9-99.5) | 99.1  (-10.4-99.5) | 98.5  (7.7-99.6) |
| 3-VAC(BNT)/COVID+ve/E | 99.4  (38-99.9) | 99.3  (33-99.6) | 99.4  (80.9-99.6) | 99.4  (9.5-99.6) | 99.3  (95.7-99.6) | 99.4  (88.3-99.6) |
| 4-VAC(non-BNT)/COVID+ve/E | 99.4  (-10.5-99.7) | 99.3  (-8.9-99.6) | 99.3  (3.4-99.6) | 99.1  (15.4-99.8) | 99.1  (82.5-99.6) | 99.1  (57.7-99.6) |
| 5-COVID+ve/E/VAC(any) | 83.3  (5.4-99.6) | 98.7  (-1.1-99.6) | 98.8  (19.3-99.6) | 87.8  (44.9-99.6) | 99.1  (8-99.6) | 99.5  (7.1-99.6) |

**Supplementary Table 2.** Kaplan-Meier survival analysis for incidence rate and median time to SARS-CoV-2 breakthrough infection following primary vaccination across various post-vaccination periods and by study groups.

*Abbreviations: SARS-CoV-2, Severe Acute Respiratory Syndrome Coronavirus 2; E, study enrollment; VAC(BNT), vaccinated with BNT162b2; VAC(non-BNT), vaccinated with a COVID-19 vaccine other than BNT162b2; VAC(any), vaccinated with any type of COVID-19 vaccine; COVID-ve, negative for COVID-19; COVID+ve, positive for COVID-19; "/" delineates sequential events in chronological order.*

| **Group** | **Number of participants**  **(n)** | **Incidence** | **Incidence rate**  **(per 1000 person per day)** | | **Median time to SARS-CoV-2 infection**  **(day)** | **Spearman correlation coefficient between incidence rate and median time to SARS-CoV-2 infection**  **(p-value)** |
| --- | --- | --- | --- | --- | --- | --- |
| ***Time to infection since the 1^st^ vaccination dose*** |  |  |  |  | |  |
| 1-E/VAC(BNT)/COVID-ve | 52 | 0 | 0 | | - | -0.97  (p=0.0024) |
| 2-E/VAC(BNT)/COVID+ve | 85 | 75 | 2.90 | | 326 |  |
| 3-VAC(BNT)/COVID+ve/E | 62 | 62 | 4.30 | | 256 |  |
| 4-VAC(non-BNT)/COVID+ve/E | 39 | 37 | 4.00 | | 231 |  |
| 5-COVID+ve/E/VAC(any) | 29 | 15 | 1.10 | | 575 |  |
| ***Time to infection since the 2^nd^ vaccination dose*** |  |  |  | |  |  |
| 1-E/VAC(BNT)/COVID-ve | 52 | 0 | 0 | | - | -0.94  (p=0.0030) |
| 2-E/VAC(BNT)/COVID+ve | 85 | 84 | 3.07 | | 304 |  |
| 3-VAC(BNT)/COVID+ve/E | 62 | 59 | 3.86 | | 251 |  |
| 4-VAC(non-BNT)/COVID+ve/E | 39 | 33 | 3.21 | | 203 |  |
| 5-COVID+ve/E/VAC(any) | 29 | 14 | 1.04 | | 554 |  |
| ***Time to infection since the day-15 after 2^nd^ vaccination dose*** |  |  |  | |  |  |
| 1-E/VAC(BNT)/COVID-ve | 52 | 0 | 0 | | - | -0.92  (p=0.0037) |
| 2-E/VAC(BNT)/COVID+ve | 85 | 84 | 3.21 | | 289 |  |
| 3-VAC(BNT)/COVID+ve/E | 62 | 59 | 4.09 | | 236 |  |
| 4-VAC(non-BNT)/COVID+ve/E | 39 | 33 | 3.24 | | 188 |  |
| 5-COVID+ve/E/VAC(any) | 29 | 15 | 1.06 | | 539 |  |

**Supplementary Table 3.** Correlation matrix of logrank significance test of Kaplan-Meier analysis for incidence rate of and time to SARS-CoV-2 breakthrough infection following complete primary vaccination (day-15 after 2^nd^ vaccination dose) across various post-vaccination periods and by study groups.

*Abbreviations: SARS-CoV-2, Severe Acute Respiratory Syndrome Coronavirus 2; E, study enrollment; VAC(BNT), vaccinated with BNT162b2; VAC(non-BNT), vaccinated with a COVID-19 vaccine other than BNT162b2; VAC(any), vaccinated with any type of COVID-19 vaccine; COVID-ve, negative for COVID-19; COVID+ve, positive for COVID-19; "/" delineates sequential events in chronological order.*

|  | 2-E/VAC(BNT)/COVID+ve | 3-VAC(BNT)/COVID+ve/E | 4-VAC(non-BNT)/COVID+ve/E | 5-COVID+ve/E/VAC(any) |
| --- | --- | --- | --- | --- |
| 2-E/VAC(BNT)/COVID+ve |  |  |  |  |
| 3-VAC(BNT)/COVID+ve/E | p = 0.055 |  |  |  |
| 4-VAC(non-BNT)/COVID+ve/E | p = 0.061 | p = 0.065 |  |  |
| 5-COVID+ve/E/VAC(any) | p < 0.001 | p < 0.001 | p < 0.001 |  |

**Supplementary Table 4.** Variations in breakthrough infection (BTI) incidence rate of 1-E/VAC(BNT)/COVID-ve and 2-E/VAC(BNT)/COVID+ve (Participants receiving BNT162b2 vaccine, n=137) based on low, moderate, and high antibody level on Day-37 post-first vaccination dose (indicating complete vaccination) and Day-90 post-first vaccination dose (representing early period of antibody waning). Antibody levels below the 25th percentile were classified as “Low,” those exceeding the 75th percentile were classified as “High,” and the intermediate range, representing the middle 50% of the distribution, was categorized as “Moderate” antibody levels. Chi-square analysis was performed between the timepoints. P-values < 0.05 were considered significant.

*Abbreviations: Ab, antibodies; BTI, breakthrough infection; NAb, neutralizing antibodies*

| **Starting timepoint** | **Day-37 post first BNT162b2 dose**  **(Complete vaccination)** | | | **Day-90 post first BNT162b2 dose**  **(Early Ab waning period)** | | | **Difference** | |
| --- | --- | --- | --- | --- | --- | --- | --- | --- |
|  | **Cumulative person time to first documented BTI**  **(day)** | **Number of participants with documented BTI** | **Incidence rate per 1000 person-time (95% CI)** | **Cumulative person time to first documented BTI**  **(day)** | **Number of participants with documented BTI** | **Incidence rate per 1000 person-time (95% CI)** | **Incidence rate difference per 1000 person-time (95% CI)** | **p-value** |
| **IgM** | | | | | | | | |
| Low | 8814 | 25 | 2.84 (1.92, 4.20) | 8487 | 24 | 2.83 (1.90, 4.22) | 0.01 (-1.58, 1.59) | 0.992 |
| Moderate | 17768 | 52 | 2.93 (2.23, 3.84) | 19442 | 52 | 2.67 (2.04, 3.51) | 0.25 (-0.83, 1.33) | 0.647 |
| High | 8922 | 25 | 2.80 (1.89, 4.15) | 7575 | 26 | 3.43 (2.34, 5.04) | -0.63 (-2.35, 1.09) | 0.472 |
| **IgA** | | | | | | | | |
| Low | 9013 | 25 | 2.77 (1.87, 4.10) | 8347 | 23 | 2.79 (1.83, 4.15) | 0.02 (-1.55, 1.58) | 0.983 |
| Moderate | 16439 | 51 | 3.10 (2.36, 4.08) | 18257 | 51 | 2.79 (2.12, 3.68) | 0.31 (-0.84, 1.45) | 0.597 |
| High | 10052 | 26 | 2.59 (1.76, 3.80) | 8900 | 28 | 3.15 (2.17, 4.56) | -0.56 (-2.09, 0.97) | 0.475 |
| **IgG** | | | | | | | | |
| Low | 9679 | 25 | 2.58 (1.75, 3.82) | 10001 | 24 | 2.40 (1.61, 3.58) | 0.18 (-1.21, 1.58) | 0.799 |
| Moderate | 15783 | 52 | 3.29 (2.51, 4.32) | 18165 | 52 | 2.86 (2.18, 3.76) | 0.43 (-0.75, 1.62) | 0.475 |
| High | 10042 | 25 | 2.49 (1.68, 3.68) | 7338 | 26 | 3.54 (2.41, 5.20) | -1.05 (-2.73, 0.62) | 0.211 |
| **NAb** | | | | | | | | |
| Low | 9383 | 25 | 2.66 (1.80, 3.94) | 9624 | 25 | 2.60 (1.76, 3.84) | 0.07 (-1.39, 1.52) | 0.929 |
| Moderate | 16773 | 52 | 3.10 (2.36, 4.07) | 18912 | 51 | 2.70 (2.05, 3.55) | 0.40 (-0.72, 1.53) | 0.481 |
| High | 9348 | 25 | 2.67 (1.81, 3.96) | 6968 | 26 | 3.73 (2.54, 5.48) | -1.06 (-2.83, 0.72) | 0.238 |
